# Continuous flow based catch and release protocol for the synthesis of α-ketoesters

**DOI:** 10.3762/bjoc.5.23

**Published:** 2009-05-20

**Authors:** Alessandro Palmieri, Steven V Ley, Anastasios Polyzos, Mark Ladlow, Ian R Baxendale

**Affiliations:** 1 Innovative Technology Centre (ACS), Department of Chemistry, University of Cambridge, Lensfield Road, Cambridge, CB2 1EW, United Kingdom; 2 CSIRO Molecular and Health Technologies, Bayview Avenue, Clayton South, Melbourne, Australia, 3169; 3 Uniqsis, Shepreth, Cambridgeshire, SG8 6GB, United Kingdom

**Keywords:** catch and release, flow synthesis, α-ketoesters, mesoreactor, polymer supported reagents

## Abstract

Using a combination of commercially available mesofluidic flow equipment and tubes packed with immobilised reagents and scavengers, a new synthesis of α-ketoesters is reported.

## Introduction

Organic synthesis is changing rapidly owing to the discovery of processes that challenge current dogma and lead to the invention of new chemical reactions [1–2]. Likewise, new synthesis tools are impacting on the way we assemble molecules. Of these, flow chemistry technologies are becoming especially important [3–14]. For many years, our group [15–22] has been focussed on using immobilised systems [23–29] to more effectively and cleanly bring about chemical transformations, especially in multistep mode [17,30–37]. Given the success of these concepts, it is not surprising that we would want to adapt these principles to various flow-chemical synthesis platforms to effectuate automated multistep chemical syntheses [38–54].

**Figure 1:**
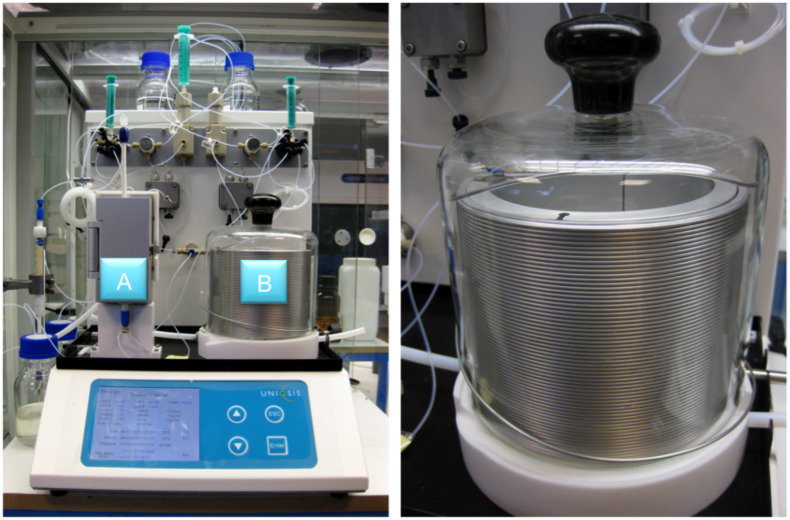
The Uniqsis FlowSyn™ continuous flow reactor comprising of a column holder and heating unit (A) and the reactor coil (B). A detailed image of the reactor coil is shown on the right.

In this work we report the use of the Uniqsis FlowSyn™ continuous flow reactor [55] (Figure 1) to effect a flow-based preparation of α-ketoesters. The key feature of this process is the application of a catch and release protocol [56–72], under the flow reaction conditions. Our choice of α-ketoesters as products of the process was governed by their use as starting materials for various synthesis programmes [73–81] and as important products in their own right [82–88]. Common methods for the preparation of α-ketoesters include the modified Dakin-West reaction [89] and the addition of a Grignard reagent to oxalates or oxalyl chlorides [90–92] together with a few alternative syntheses [93–99]. These procedures often suffer from drastic conditions, restricted selectivity and poor yields. Our flow-based approach delivers a new and general method for the preparation of α-ketoesters, which proceeds under mild conditions, with good functional group tolerability and generates products in high purity.

## Results and Discussion

The experimental set up for these transformations involves the use of the Uniqsis FlowSyn™ device [55]. The fully integrated instrument employs a dual channel flow system, with each channel independently driven by a variable high-pressure piston pump. The starting materials and reagents are dispensed from sample loops (0.5–10 mL) and are united in a T-mixing piece and then passed into either a coil or column reactor (Figure 1). The column reactor utilises adjustable glass columns with variable internal diameter (1–1.5 cm) and range in volume from 6–83 mL (unpacked). The coil reactors are made from a selection of materials including PTFE, PEEK, stainless steel or Hastelloy^®^ and accommodate volumes from 2–20 mL. The column reactor (Figure 1, A) can be heated up to 150 °C and the coil heater (Figure 1, B) up to 260 °C, over a range of flow rates between 0.01–20 mL/min, and can be configured for multistep or parallel operation. Exiting products can be collected as aliquots using an automated fraction collector for reaction optimisation or as a bulk sample for scale-up. In addition, product purification can be achieved as part of the overall flow process by in-line solid phase extraction (SPE) or alternatively by diverting the flow stream into an attached HPLC system [100].

**Scheme 1:**
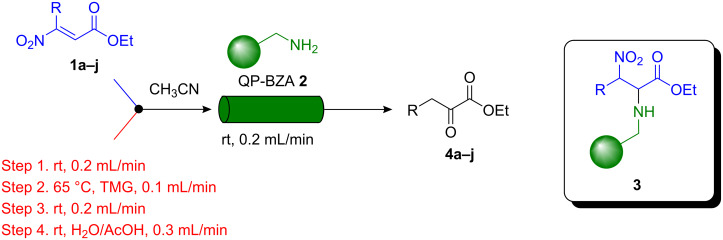
General procedure for the flow synthesis of α-ketoester products **4a**–**j**.

A series of preliminary experiments was carried out on the flow equipment to profile the reaction in terms of optimum reaction temperature, concentration, residence time, solvent and stoichiometry. Following rapid screening of conditions, we fixed upon a set of reaction parameters for efficient synthesis of α-ketoesters (Scheme 1). The overall reaction process proceeds in the flow apparatus *via* nitroolefinic esters **1** as substrates which are captured onto a benzylamine polymer **2** (QuadraPure™ QP-BZA polymer, loading 5.5 mmol/g, 4 equiv) to give **3** to effect product clean-up. In this way the immobilised species **3** can be washed and any solution phase impurities (resulting from the formation of the nitroolefinic ester – see later) are directed to waste (step 1). Next the column is treated with a flow stream of tetramethylguanidine (TMG; step 2) to cause elimination of nitrous acid and produce the corresponding enamino acid esters, which remain attached to the polymer support. Finally, after flow-washing (step 3), the solid supported species is hydrolysed, liberating α-ketoester product **4** by flowing aqueous acetic acid (step 4) through the in-line column. The overall route constitutes a new flow chemistry example of the *catch*-*react*-*and*-*release* concept that we have used successfully in other synthesis programmes [101–103].

**Scheme 2:**
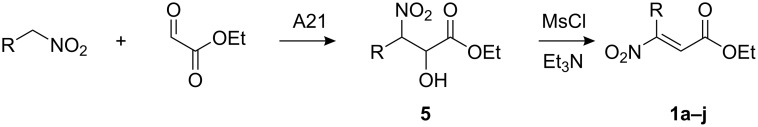
General procedure for the batch synthesis of nitroolefinic esters **1a**–**j**.

The nitroolefinic esters **1** were originally formed in a separate batch reaction from a Henry coupling of appropriate nitro compounds with ethyl glyoxalate over Amberlyst™ 21 (A21) resin to give the corresponding nitroalkanol **5** [104]. This was followed by treatment of **5** with methanesulfonyl chloride (MsCl) or trifluoroacetic anhydride (TFAA) to promote the base-catalysed dehydration, affording the nitroolefinic esters **1** (Scheme 2) [105]. As we have deliberately constructed this sequence for implementation in a continuous flow process, the intermediate nitroalkanols **5** were not isolated and the nitroolefinic esters were used without further purification. The average yield for the nitroolefins **1a**–**j** prepared as described in Scheme 2 was approximately 60% by LCMS. Impurities were readily removed following immobilisation of nitroolefinic esters **1** on the QP-BZA resin.

**Scheme 3:**
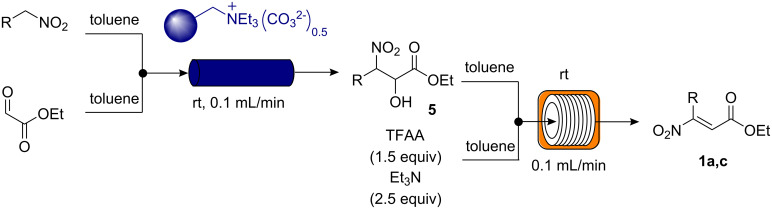
General procedure for the flow synthesis of nitroolefinic esters **1a**,**c**.

In addition, the flow synthesis of two representative compounds was undertaken to allow for the complete generation of α-ketoester products in flow from the starting nitroalkanes (Scheme 3). As shown in Table 1, we demonstrate that the synthesis of the nitroolefinic esters was achieved under flow conditions in a clean and effective fashion. Moreover, this synthesis demonstrates the first reported example of Henry reaction conducted in flow and we intend to elaborate on this important transformation in future studies.

**Table 1:** Nitroolefinic esters **1a,c** prepared under flow conditions (as described in Scheme 3).

Entry	**1**	Compound	Yield (%)^a^

1	**1a**	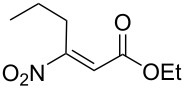	63
2	**1c**	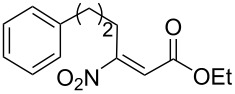	55

^a^Isolated yields are shown.

**Figure 2:**
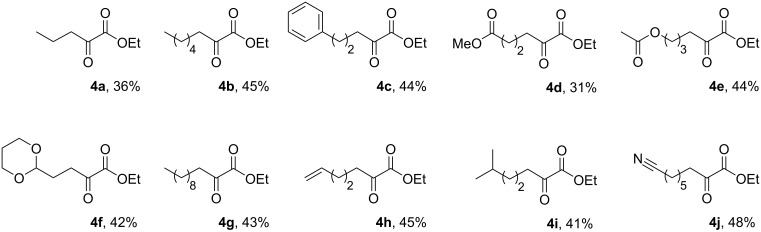
α-Ketoesters prepared and isolated yields.

Figure 2 illustrates the examples and yields of α-ketoester products afforded by this new approach. While the list is not extensive, we have established that the process is tolerant of both aliphatic and aromatic substituted nitro-derivatives in the first step, and accommodates ester, acetate, acetal, nitrile and olefinic functionality in the final product. The process was reliable over several runs and consistently afforded very clean material (≥ 97% by NMR). The yields while only moderate for the *overall process* still equate to an average step conversion of 68–78% per chemical iteration, given that the sequence is a multistep process (see Supporting Information for full experimental data).

## Conclusion

In conclusion, we have demonstrated the versatility of the Uniqsis FlowSyn™ unit to achieve multi-step organic synthesis under continuous flow-chemistry conditions. This was accomplished by adapting the device to incorporate immobilised reagents packed in flow tubes, enabling clean transformations without recourse to conventional product work-up or purification. The overall process delivers synthetically useful α-ketoester products in high purity from various nitroalkane inputs and paves the way for more extended reaction sequences.

## Supporting Information

File 1 Supporting Information – Continuous flow based catch and release protocol for the synthesis of α-ketoesters
