# Supplementary material for: Continuous flow based catch and release protocol for the synthesis of α-ketoesters
Source: Beilstein J Org Chem. 2009 May 20;5:23. doi: 10.3762/bjoc.5.23 (PMC2707014; doi:10.3762/bjoc.5.23)
Supplement: File 1 — Supporting Information – Continuous flow based catch and release protocol for the synthesis of α-ketoesters [file Beilstein_J_Org_Chem-05-23-s001.doc]

# Supporting Information

# Continuous flow based catch and release protocol for the synthesis of α‑ketoesters

Alessandro Palmieri^1^, Steven V. Ley*^,1^, Anastasios Polyzos^1,2^, Mark Ladlow^3^ and
Ian R. Baxendale^1^

Address: ^1^Innovative Technology Centre (ACS), Department of Chemistry, University of Cambridge, Lensfield Road, Cambridge, CB2 1EW, United Kingdom;

^2^CSIRO Molecular and Health Technologies, Bayview Avenue, Clayton South, Melbourne, Australia, 3169 and

^3^Uniqsis, Shepreth, Cambridgeshire, SG8 6GB, United Kingdom

Email: Steven V. Ley - svl1000@cam.ac.uk

* Corresponding author

**General**

^1^H NMR spectra were recorded on a Bruker Avance DPX-400 or DRX-500 spectrometer with residual chloroform as the internal reference (CHCl_3_ *δ*_H_ = 7.26 ppm).^13^C NMR spectra were also recorded in CDCl_3_ on the same spectrometer with the central peak of the residual solvent as the internal reference (*δ*_C_ = 77.0 ppm). Infrared spectra were recorded on a Perkin-Elmer Spectrum One FT-IR spectrometer. HRMS analysis was performed on a LCT Premier Micromass spectrometer.

**General Synthesis of Nitroolefinic esters in Flow**

A solution of the appropriate nitroalkane (1.0 mmol) in toluene (10 mL) and ethyl glyoxylate (1.0 mmol) in toluene (10 mL) were each injected into a separate sample loop of the Uniqsis instrument. The valve was then set to inject and the material was passed through an Omnifit® column at room temperature containing 0.36 g of MP-Carbonate polymer (loading 2.8 mmol/g, 1 equiv) using toluene as a solvent and flow rate of 0.1 mL/min. This product stream was collected, dried with Na_2_SO_4_ and the solvent was removed under vacuum to afford the crude nitroalkanol **5**. Then two solutions were prepared, one containing the nitroalkanol **5** in toluene (1.0 mmol, 25 mL) and the other containing TFAA (1.5 mmol) and Et_3_N (2.5 mmol) in 25 mL toluene. The sample loops were then filled with each solution. After the samples loops were set to inject, the nitroalkanol substrate flow stream, the TFAA and Et_3_N reagent streams were then united in a T-piece mixer and passed through the Uniqsis 20 mL stainless steel reactor coil at a flow rate of 0.1 mL/min, at room temperature. This product stream was collected directly into a separatory funnel. The organic layer in the separatory funnel was treated with HCl (2 m) and the organic layer then separated, dried with Na_2_SO_4_. The solvent was removed under vacuum to afford nitroolefinic ester **1**, which was then purified by column chromatography (hexane/EtOAc, 95 : 5, on SiO_2_).

**(*E*)-Ethyl 3-nitropent-2-enoate (1a)** [1]. Yield = 63%. IR (neat),*ν* (%): 2984 (51), 1728 (98), 1670 (42), 1538 (100), 1342 (78), 1309 (79), 1200 (95), 1042 (71) cm^−1^. ^1^H NMR (400 MHz, CDCl_3_) *δ*: 1.18 (t, 3H, *J* = 7.7 Hz), 1.33 (t, 3H, *J* = 7.3 Hz), 3.06 (q, 2H, *J* = 7.3 Hz), 4.27 (q, 2H, *J* = 7.3 Hz), 6.96 (s, 1H).^13^C NMR (100 MHz, CDCl_3_) *δ*: 12.53, 14.23, 21.24, 61.93, 120.80, 164.15, 165.43.

**(*E*)-Ethyl 3-nitro-5-phenylpent-2-enoate (1c)**.Yield = 55%. IR (neat),*ν* (%): 3028 (64), 2939 (71), 1726 (98), 1670 (67), 1537 (100), 1211 (91), 1173 (89), 1030 (82), 902 (67), 860 (64), 750 (75), 700 (84) cm^−1^. ^1^H NMR (400 MHz, CDCl_3_) *δ*: 1.31 (t, 3H, *J* = 7.3 Hz), 2.84–2.91 (m, 2H), 3.33–3.39 (m, 2H), 4.22 (q, 2H, *J* = 7.3 Hz), 7.02 (s, 1H), 7.20–7.25 (m, 3H), 7.27–7.33 (m, 2H).^13^C NMR (100 MHz, CDCl_3_) *δ*: 14.24, 29.61, 34.31, 61.98, 122.08, 126.74, 128.73, 128.81, 139.83, 162.84, 164.01.

**General Synthesis of α-Ketoesters in Flow**

A solution of nitroolefin **1** (1.5 mmol) in MeCN (10 mL) was injected into the sample loop of the Uniqsis instrument. The valve was set to inject and the material was passed through a column containing 1.090 g of QP-BZA polymer (loading 5.5 mmol/g, 4 equiv) using MeCN at a solvent flow rate of 0.2 mL/min at room temperature. Then the QP-BZA polymer was heated to 65 °C using the column heater unit on the Uniqsis. A solution of tetramethylguanidine (3 mmol) in MeCN (10 mL) was injected into the second sample loop and the material was then passed through the column using MeCN at a flow rate of 0.1 mL/min. The QP-BZA polymer was then washed with 20 mL of MeCN at solvent flow rate of 1 mL/min to remove any impurities. The corresponding immobilized enamino acid ester was then hydrolyzed with the injection of a water/acetic acid solution (9 : 1, 15 mL) using MeCN as a solvent at a flow rate of 0.3 mL/min. This product stream was collected directly into a separatory funnel. Then a stream of EtOAc (40 mL) was flowed through the QP-BZA polymer column at a flow rate of 1 mL/min to extract the residual ketoester **1**, which was collected directly into the same separatory funnel. The organic layer was separated and the aqueous layer extracted with EtOAc (2 × 25 mL). The organic extracts were combined and then dried with Na_2_SO_4_. The solvent was removed under vacuum to afford ketoester **4**, which was isolated with sufficient purity to avoid subsequent purification steps.

**Ethyl 2-oxopentanoate (4a)** [2]. Yield = 36%; IR (neat), *ν* (%): 2968 (22), 1725 (100), 1465 (19), 1257 (60), 1116 (51), 1052 (74) cm^−1^. ^1^H NMR (400 MHz, CDCl_3_) *δ*: 0.95 (t, 3H, *J* = 7.3 Hz), 1.35 (t, 3H, *J* = 7.3 Hz), 1.59–1.71 (m, 2H), 2.79 (t, 2H, *J* = 7.3 Hz), 4.30 (q, 2H, *J* = 7.3 Hz). ^13^C NMR (100 MHz, CDCl_3_) *δ*: 13.48, 13.99, 16.52, 41.11, 62.30, 161.34, 194.65.

**Ethyl 2-oxooctanoate (4b)** [3]. Yield = 45%; IR (neat), *ν* (%): 2931 (34), 1726 (100), 1467 (20), 1263 (48), 1124 (32), 1062 (77) cm^−1^. ^1^H NMR (400 MHz, CDCl_3_) *δ*: 0.88 (t, 3H, *J* = 6.7 Hz), 1.24–1.39 (m, 9H), 1.57–1.68 (m, 2H), 2.81 (t, 2H, *J* = 7.3 Hz), 4.31 (q, 2H, *J* = 7.3 Hz). ^13^C NMR (100 MHz, CDCl_3_) *δ*: 14.34, 14.38, 22.80, 23.35, 29.01, 31.84, 39.67, 62.70, 161.76, 195.21.

**Ethyl 2-oxo-5-phenylpentanoate (4c)** [4].Yield = 44%; IR (neat), *ν* (%): 3027 (11), 2937 (17), 1725 (100), 1604 (10), 1454 (32), 1261 (51), 1071 (56) cm^−1^. ^1^H NMR (400 MHz, CDCl_3_) *δ*: 1.36 (t, 3H, *J* = 7.3 Hz), 1.93–2.04 (m, 2H), 2.67 (t, 2H, *J* = 7.3 Hz), 2.85 (t, 2H, *J* = 7.3 Hz), 4.30 (q, 2H, *J* = 7.2 Hz), 7.14-7.23 (m, 3H), 7.24-7.32 (m, 2H). ^13^C NMR (100 MHz, CDCl_3_) *δ*: 14.03, 24.58, 34.87, 38.55, 62.41, 126.14, 128.50, 141.16, 161.22, 194.43.

**1-Ethyl 6-methyl 2-oxohexanedioate (4d)**. Yield = 31%; IR (neat), *ν* (%): 2954 (14), 1726 (100), 1438 (31), 1247 (59), 1200 (55), 1175 (52), 1086 (54), 1041 (56) cm^−1^. ^1^H NMR (400 MHz, CDCl_3_) *δ*: 1.36 (t, 3H, *J* = 7.3 Hz), 1.91–2.01 (m, 2H), 2.38 (t, 2H, *J* = 7.3 Hz), 2.92 (t, 2H, *J* = 7.3 Hz), 3.67 (s, 3H), 4.31 (q, 2H, *J* = 7.3 Hz). ^13^C NMR (100 MHz, CDCl_3_) *δ*: 13.99, 18.21, 32.70, 38.30, 51.63, 62.48, 160.96, 173.25, 193.74.

**Ethyl 6-acetoxy-2-oxohexanoate (4e)**. Yield = 44%; IR (neat), *ν* (%): 2959 (12), 1725 (100), 1367 (34), 1235 (95), 1038 (75) cm^−1^. ^1^H NMR (400 MHz, CDCl_3_) *δ*: 1.36 (t, 3H, *J* = 7.3 Hz), 1.61–1.77 (m, 4H), 2.03 (s, 3H), 2.87 (t, 2H, *J* = 6.6 Hz), 4.07 (t, 2H, *J* = 6.1 Hz), 4.31 (q, 2H, *J* = 7.3 Hz). ^13^C NMR (100 MHz, CDCl_3_) *δ*: 13.99, 19.52, 20.91, 27.83, 38.68, 62.45, 63.88, 161.12, 171.09, 194.10.

**Ethyl 4-(1,3-dioxan-2-yl)-2-oxobutanoate (4f)**. Yield = 42%; IR (neat), *ν* (%): 2967 (25), 2853 (27), 1725 (100), 1470 (23), 1377 (35), 1280 (46), 1244 (72), 1146 (84), 1133 (92), 1087 (75), 1037 (97), 1005 (77) cm^−1^. ^1^H NMR (400 MHz, CDCl_3_) *δ*: 1.24–1.42 (m, 4H), 1.92–2.12 (m, 3H), 2.95 (t, 2H, *J* = 7.0 Hz), 3.72 (t, 2H, *J* = 12.1 Hz), 4.01–4.11 (m, 2H), 4.31 (q, 2H, *J* = 7.3 Hz), 4.58–4.63 (m, 1H). ^13^C NMR (100 MHz, CDCl_3_) *δ*: 14.03, 25.61, 29.07, 33.56, 62.25, 66.74, 100.29, 161.05, 194.01. HRMS Calcd. C_10_H_18_O_5_[(M + H)^+^] 217.1071, found 217.1073.

**Ethyl 2-oxododecanoate (4g)** [5]. Yield = 43%; IR (neat), *ν* (%): 2923 (67), 2854 (48), 1728 (100), 1510 (40), 1467 (37), 1303 (66), 1251 (63), 1111 (48), 1051 (62) cm^−1^. ^1^H NMR (400 MHz, CDCl_3_) *δ*: 0.88 (t, 3H, *J* = 6.2 Hz), 1.19–1.41 (m, 17H), 1.56–1.68 (m, 2H), 2.82 (t, 2H, *J* = 7.3 Hz), 4.31 (q, 2H, *J* = 7.3 Hz). ^13^C NMR (100 MHz, CDCl_3_) *δ*: 14.01, 14.09, 22.67, 23.02, 28.98, 29.29, 29.41, 29.53, 31.89, 39.30, 62.32, 161.38, 194.84.

**Ethyl 2-oxohept-6-enoate (4h)** [6]. Yield = 45%;IR (neat), *ν* (%): 3080 (4), 2939 (14), 1726 (100), 1641 (14), 1446 (17), 1266 (50), 1070 (55), 1041 (57), 914 (45) cm^−1^. ^1^H NMR (400 MHz, CDCl_3_) *δ*: 1.36 (t, 3H, *J* = 7.3 Hz), 1.69–1.80 (m, 2H), 2.04–2.15 (m, 2H), 2.84 (t, 2H, *J* = 7.3 Hz), 4.31 (q, 2H, *J* = 7.3 Hz), 4.94–5.08 (m, 2H), 5.67–5.84 (m, 1H). ^13^C NMR (100 MHz, CDCl_3_) *δ*: 14.00, 22.13, 32.79, 38.45, 62.37, 115.65, 137.50, 161.25, 194.53.

**Ethyl 6-methyl-2-oxoheptanoate (4i)**. Yield = 41%; IR (neat), *ν* (%): 2956 (36), 1726 (100), 1469 (27), 1368 (24), 1266 (48), 1238 (50), 1132 (31), 1067 (71), 1045 (57) cm^−1^. ^1^H NMR (400 MHz, CDCl_3_) *δ*: 0.87 (d, 6H, *J* = 6.6 Hz), 1.16–1.23 (m, 2H), 1.36 (t, 3H, *J* = 7.1 Hz), 1.50–1.58 (m, 1H), 1.59–1.66 (m, 2H), 2.80 (t, 2H, *J* = 7.1 Hz), 4.30 (q, 2H, *J* = 7.1 Hz). ^13^C NMR (100 MHz, CDCl_3_) *δ*: 13.96, 20.84, 22.36, 27.71, 38.11, 39.45, 62.28, 161.29, 194.77.

**Ethyl 8-cyano-2-oxooctanoate (2j)**. Yield = 48%; IR (neat), *ν* (%): 2939 (27), 2245 (9), 1725 (100), 1464 (20), 1269 (46), 1244 (51), 1097 (43), 1055 (72) cm^−1^. ^1^H NMR (400 MHz, CDCl_3_) *δ*: 1.31–1.54 (m, 7H), 1.59–1.73 (m, 4H), 2.34 (t, 2H, *J* = 7.3 Hz), 2.84 (t, 2H, *J* = 7.3 Hz), 4.31 (q, 2H, *J* = 7.3 Hz). ^13^C NMR (100 MHz, CDCl_3_) *δ*: 14.01, 17.07, 22.59, 25.14, 28.06, 28.34, 38.99, 62.44, 119.60, 161.21.

**References**

1. Ballini, R.; Fiorini, D.; Palmieri, A. *Tetrahedron Lett.* **2004,** *45,* 7027–7029. doi:[10.1016/j.tetlet.2004.07.141](http://dx.doi.org/10.1016/j.tetlet.2004.07.141)
2. Nakamura, K.; Inoue, K.; Ushio, K.; Oka, S.; Ohno, A. *J. Org. Chem.* **1988,** *53,* 2589–2593. doi:[10.1021/jo00246a035](http://dx.doi.org/10.1021/jo00246a035)
3. Bergeron, R. J.; Hoffman, P. G. *J. Org. Chem.* **1979,** *44,* 1835–1839. doi:[10.1021/jo01325a019](http://dx.doi.org/10.1021/jo01325a019)
4. Weinstock, L. M.; Currie, R. B.; Lovell, A. V. *Synth. Commun.* **1981,** *11,* 943–946. doi:[10.1080/00397918108065753](http://dx.doi.org/10.1080/00397918108065753)
5. Rozen, S.; Ben-David, I. *J. Org. Chem.* **2001,** *66,* 496–500. doi:[10.1021/jo0013094](http://dx.doi.org/10.1021/jo0013094)
6. Macritchie, J. A.; Silcock, A.; Willis, C. L. *Tetrahedron: Asymmetry* **1997,** *8,* 3895–3902. doi:[10.1016/S0957-4166(97)00571-5](http://dx.doi.org/10.1016/S0957-4166(97)00571-5)
